# Augmentation of plant biomass productivity using epigeic earthworm *Perionyx excavatus* and *Eisenia fetida* as soil nutrient facilitators

**DOI:** 10.1038/s41598-023-45288-9

**Published:** 2023-10-30

**Authors:** Lirikum Jing, Lakhmi Nandan Kakati, Bendang Ao, Patricia Kiewhuo

**Affiliations:** 1 Department of Zoology, Don Bosco College, Kohima, Nagaland India; 2 Department of Zoology, Nagaland University, Lumami, Zunheboto, Nagaland India; 3 Faculty of Science, Assam Down Town University, Panikhaiti, Guwahati-26, Guwahati, Assam India

**Keywords:** Plant sciences, Zoology, Environmental sciences

## Abstract

With the increasing demand for organic food production, the earthworm is used as a soil nutrient facilitator. The present study was conducted to assess the effect of epigeic earthworms *Perionyx excavatus* and *Eisenia. fetida* on soil nutrients and the consequent improvement of biomass productivity and yield of *Capsicum chinense* Jacq and *Zea mays* L*.* The experiment was conducted in 5 L and 15 L capacity plastic pots for *C. chinense* and *Z. mays* with 150 g and 300 g of half-decomposed cow dung, respectively. It was observed that the weekly harvest rate of ripened chili was 17.59 g, 13.91 g, and 9.24 g in *P. excavatus*, control, and *E. fetida* pot showing 26.49% higher in *P. excavatus*. Also, the total kernel count per corn was significantly different (*F*_(2, 9)_ = 37.78, *p* < 0.05), with the highest kernel present in *P. excavatus*(333.5 ± 13.5), followed by *E. fetida*(261.5 ± 16.5) and control (235 ± 22). The impact of *P. excavatus* was more perceptible in *C. chinense*, indicated by higher leaf biomass (69.16%), root length (30.14%), and fruit harvest (71.03%). However, the effect of *E. fetida* was noticed more in *Z. mays* (stem length, 19.24%, stem biomass, 14.39%, root biomass, 20.9%, kernel count, 41.91%, and kernel weight, 95.07%). Enhanced plant productivity was also supported by an increasing soil nutrient turnover in organic carbon (OC) (25.76% and 23.4%), Phosphorus (P) (31.03% and 25.67%), and Potassium (K) (41.67% and 12.26) in *P. excavatus* and *E. fetida* worked soil respectively. The findings indicate that earthworms have a notable impact on plant biomass productivity by promoting the mineralization of soil nutrients and imply on possibility of organic cultivation of seasonal vegetables without using synthetic fertilizers.

## Introduction

The rapid increase in the world's population and unprecedented human consumption necessitates increased food production. This leads to the intensive use of chemical fertilizers and pesticides in agricultural practices. These unsustainable practices to maximize productivity result in land degradation, cause human health hazards, interrupt ecological nutrient cycling, and destroy healthy biotic communities^1^. Therefore, it is vital to emphasize on cost-effective and eco-friendly methods of agriculture for a healthier future.

In soil, large-bodied macroinvertebrates such as earthworms play a crucial role in plant productivity. Through ingestion and microbial priming activities, solubilization of an inaccessible form of soil nutrients, making it available for plants, is anchored by earthworms^2–4^. Burrowing activities of earthworms aid aerations and improve biochemical properties providing stability and resilience to the soil ecosystem^5,6^. Earthworms can process up to 250 tons per hectare of soil yearly^7^ and provide optimum soil conditions for plant growth. Earthworms also increase soil organic carbon by incorporating organic materials into the soil^8^ and generate macropores that increase the water flow, which protects the soil surface against erosion^9^. With proliferative reproduction, the earthworm is an economically affordable, environmentally sustainable, and socially acceptable potential candidate for improving soil properties for better plant productivity^10^.

It is well understood that in the natural environment, different ecological categories of earthworms (epigeic, endogeic, and anecic) and their interactions with other soil biota improve the soil's physicochemical properties. Earthworms initiate numerous mechanisms for plant growth stimulation ranging from large-scale effects on soil physical properties to the microsite level. The potential effects of earthworms on ecosystem modification, plant community composition, and productivity are well demonstrated when earthworms are introduced to areas that were previously earthworm-free^11,12^. The benefits of earthworms are highlighted through increased levels of microbial activity, nutrient availability, and rhizosphere processes^13,14^. With the introduction of earthworms in soil with sandy textures, poor in organic matter, and with a moderately acidic pH, shoot and grain biomass increases to 56.3% and 35.8%, respectively^15^.

Studying earthworms’ influence on soil properties is essential to develop management strategies for improving soil fertility and plant growth in different subsystems of tropical areas. Although positive effects of earthworms on plant growth have been described in agroecosystems^16,17^, quantitative studies on the role of earthworms in augmenting plant biomass productivity have not been satisfactorily established. In a tropical country like India, earthworm species such as *Pontoscolex corethrurus* and *Drawida willsi* with biomass of around 30 g m^-2^ or more, are considered promising in plant growth and shown to increase the grain yield (> 40%) of agriculturally important plants^16^. While single species of earthworms under laboratory conditions resulted in more significant improvements in soil physico-hydraulic properties. However, such studies on the most important horticultural plants such as *Capsicum chinense* (King chili) and *Zea mays* are lacking.

*C. chinense* (King chili)*,* one of the hottest chili in the world, is commonly grown in Indian states of Assam, Manipur, and Nagaland; and besides being appreciated for its taste and pungency, it is also rich in vitamins, minerals, and nutrients^18^. King chili is one of the key ingredients in Naga cuisine and plays a vital role in the region's culinary traditions and cultural identity. The state has favorable climatic conditions for growing this chili variety, and the demand for it has also increased in recent years. Therefore employing efficient means of farming could provide windows of opportunities for entrepreneurs to gain significant economic importance. *Z. mays* (Corn, locally known as Makai) is a vital staple food crop in Nagaland and Northeast India. It plays a crucial role in the region's food security and sustains a large population. Corn-based dishes are integral to traditional cuisine, and their cultivation and consumption have deep cultural and social significance. Corn adds dietary diversity and nutritional value to the local diet with a rich amount of carbohydrates, dietary fiber, vitamins (such as thiamine and niacin), and minerals (such as phosphorus and magnesium). Also, corn cultivation is a vital livelihood source for many farmers in Nagaland. It provides employment opportunities, especially for small-scale farmers, and contributes significantly to the rural economy. The sale of corn and its by-products, such as corn flour, corn flakes, and corn oil, generates income for farmers and local entrepreneurs.Thus it is necessary to identify the design of agricultural strategies that facilitate an increase in the growth and yield of these economically potential plants while conserving the concept of organic farming and sustainability.

*P. excavatus* and *E. fetida* are the most promising earthworm species used for vermitechnology. Being epigeic, these macroinvertebrates dwell in the organic carbon-rich soil and play a vital role in nutrient turnover, heavy metal detoxification, waste biomass degradation and recycling of coal ash^19–21^. Also, apart from a few studies supporting the usage of *P. excavatus* and *E. fetida* in vermicomposting^22,23^, information on plant growth, productivity, and soil macronutrient enhancement is lacking. Therefore to abide by the initiatives to implement the practice of organic farming and sustainability, exploitation of these potential macroinvertebrates (*E. fetida* and *P. excavatus*) is vital.

Nagaland (Northeast India) is a hilly state where indigenous people depend on agricultural practices for their sustenance. There is a need to further encourage organic farming practices without subsiding the quality and quantity of productivity for viable and healthier living. In this context, the application of epigeic earthworm species like *P. excavatus* and *E. fetida* is considered sustainable, especially for *C. chinense* (Naga king chili) and *Z. mays*, popularly used by local inhabitants for consumption as well as vending in the local market. Therefore, the present study focuses on improving the biomass and yield of *C. chinense* and *Z. mays* by inoculating *P. excavatus* and *E. fetida* in the soil. We hypothesize that earthworms act as soil nutrient facilitators by acting as critical agents in nutrient turnover and enhancing the plants' growth and productivity.

## Results

### Comparative study of the effects of *P. excavatus* and *E. fetida* on *C. chinense*

**Figure 1:**
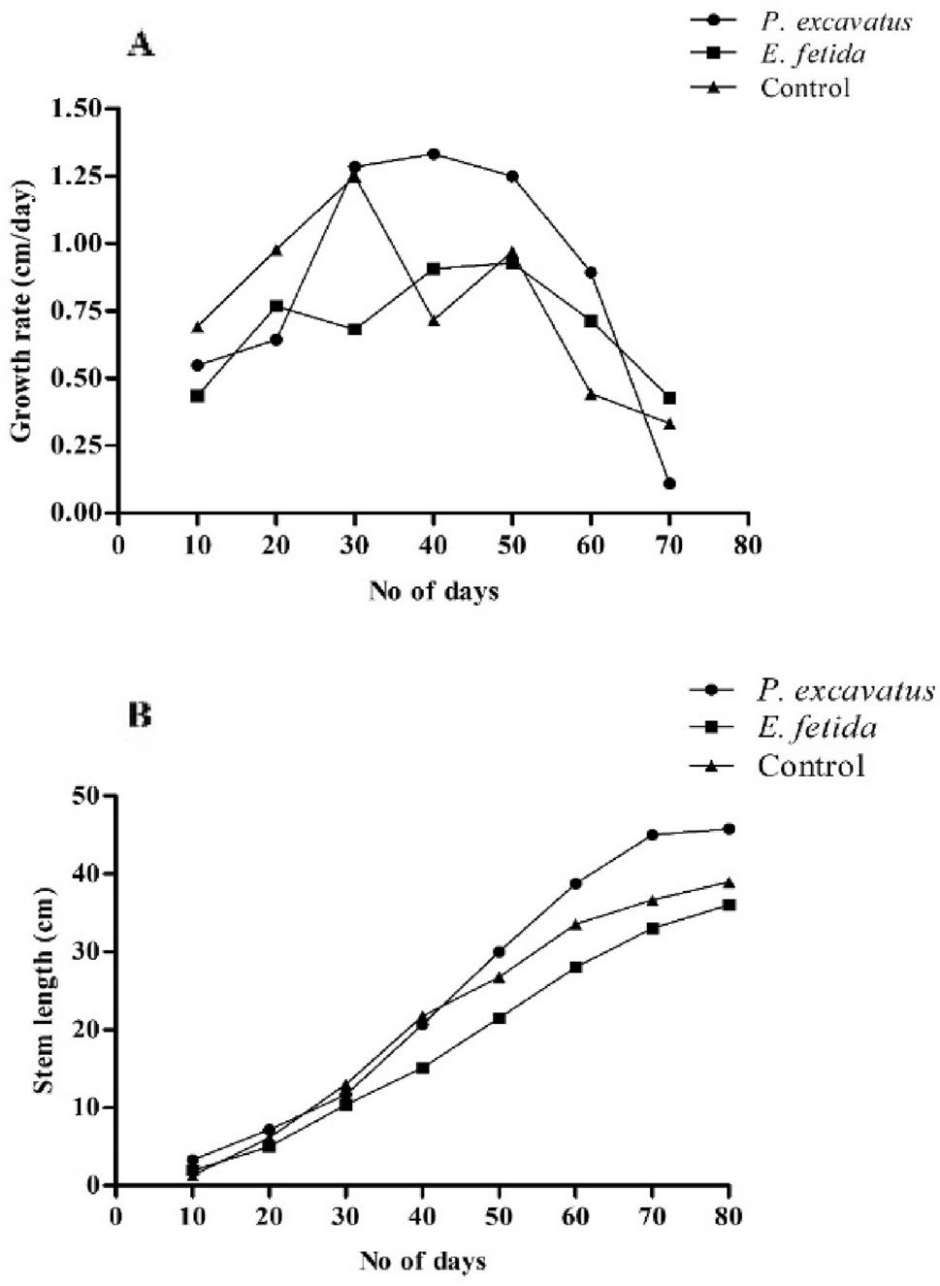
(**A**) Growth rate of *C. chinense* under earthworm treatment and control (**B**) Increasing pattern of *C. chinense*stem under earthworm-mediated soil and control.

**Figure 2:**
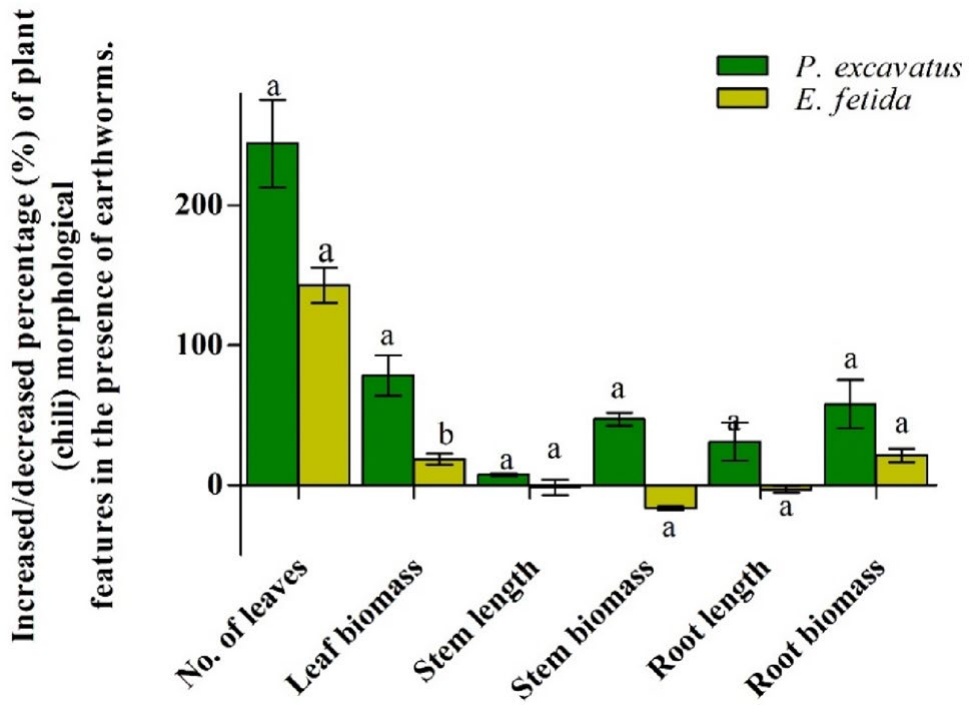
Increased/ decrease in *C. chinense* morphological characters in the presence of *P. excavatus* and *E. fetida***.** Data represent mean ± SD. Different superscripts between earthworm treatments differ significantly (*p* < 0.05) by the Tukey test at a 95% confidence level.

In *P. excavatus* inoculated soil, the average growth rate (mm/day) was maximum (8.3 ± 2), followed by the control and *E. fetida* inoculated soil showing 7.5 ± 3.5 and 5.7 ± 1.4 respectively (Fig. 1A). The average growth rate in *P. excavatus* inoculated soil was 12.59% higher than the control, while in *E. fetida* inoculated soil, it was 9.65% lower than the control. A significant difference in the number of leaves recorded from earthworm inoculated soil (163.66 ± 43.88 and 91.25 ± 13.43 in *P. excavatus* and *E. fetida* respectively), and control (46.5 ± 19.94) (*F*_(2, 9)_ = 8.9, *p* < 0.05) was recorded. A high percentage in the number of leaves was recorded in both *P. excavatus*, and *E. fetida* inoculated soils (250.40% and 142.85%, respectively). Mean leaf biomass (g) also varies significantly (*F*_(2, 12)_ = 7.89, *p* < 0.05) depending on the treatment, and maximum biomass resulted from *P. excavatus* (69.16 ± 20.73) > *E. fetida* (39.46 ± 6.7) > control (35.85 ± 6.77). With 92% and 10.06% increase over control, biomass increase in *P. excavatus* was significantly (*p* < 0.05) higher. The average stem length (mm) at the time of harvest was higher in *P. excavatus* (410 ± 49.7) than in control (398.7 ± 70.2) and *E. fetida* (368.7 ± 36.6) (Fig. 1B), showing an increase and decrease of 7.66% and 2.77% respectively over the control. Stem length was substantially higher in the presence of *P. excavatus* (Table 1). While stem biomass (g) from *P. excavatus* inoculated soil was increased by 47.30% (89.32 ± 24.27) over the control (63.00 ± 26.78), while in *E. fetida* (46.42 ± 21.27), it was decreased by 16.43% (Fig. 2).

**Table 1:** Morphological characteristics of *C. chinense* in the presence and absence of earthworms.

Treatment	No. of leaves	Leaf biomass (g)	Stem length(mm)	Stem biomass(g)	Root length (mm)	Root biomass(g)
*P. excavatus*	163.66 ± 43.88^a^	69.16 ± 20.73^a^	41 ± 4.97^a^	89.32 ± 24.27^a^	40.5 ± 8.21^a^	41.8 ± 12.75^a^
*E. fetida*	91.25 ± 13.43^ab^	28.87 ± 0.82^c^	36.87 ± 3.66^a^	46.42 ± 21.27^b^	25.12 ± 2.01^b^	31.39 ± 12.7^a^
Control	46.5 ± 19.94^b^	35.85 ± ^bc^	39.87 ± 7.02^a^	63.00 ± 26.78^a^	30.00 ± 8.04^ab^	29.2 ± 8.87^a^
*H*	8.95	7.89	0.26	2.68	8.12	2.55
*p*-value	0.007**	0.006*	0.87	0.12	0.01*	0.11

Data represent mean ± SD. * indicate a significant difference at *p* < 0.05. Mean with different superscripts within the same row differ significantly (*p* < 0.05) by the Tukey test at a 95% confidence level.

Root length (mm) in *P. excavatus* (405.00 ± 82.1), *E. fetida* (251.2 ± 20.1), and control (300.0 ± 80.4) differ significantly (*F*(_2, 9_) = 8.12, *p* < 0.05). Due to fungal infection, the root length from *E. fetida* inoculated soil was decreased by 2.42%, while in *P. excavatus,* the root length was 30.14% higher. Also, root biomass (g) was recorded to be maximum in *P. excavatus* (41.8 ± 12.75) with a 58.04% increase over control, while in *E. fetida* (31.39 ± 12.7), it was increased by 20.22% (Fig. 2). Along with the plant morphological characters, significant variations (*F*_(2, 10)_ = 5.24, *p* < 0.05) in total fruit yield (g) per plant were also recorded. Total harvest was observed to be maximum in *P. excavatus* (573.27 g), followed by control (266.8 g) and *E. fetida* (112.99 g). In *P. excavatus* inoculated soil, ripened chili was harvested at 17.59 g per week with an average of 108.13 ± 37.93 g per plant. In control and *E. fetida*, ripened chili was harvested at 13.91 g and 9.24 g per week. While the quantity of harvest per week does not differ significantly (*F*_(2, 18)_ = 1.21, *p* > 0.05) among the treatments, it was 26.49% higher (*P. excavatus)* and 33.53% lesser (*E. fetida)* over the control.

### Comparative study of theeffectsof *P. excavatus* and *E. fetida* on *Z. mays*

**Figure 3:**
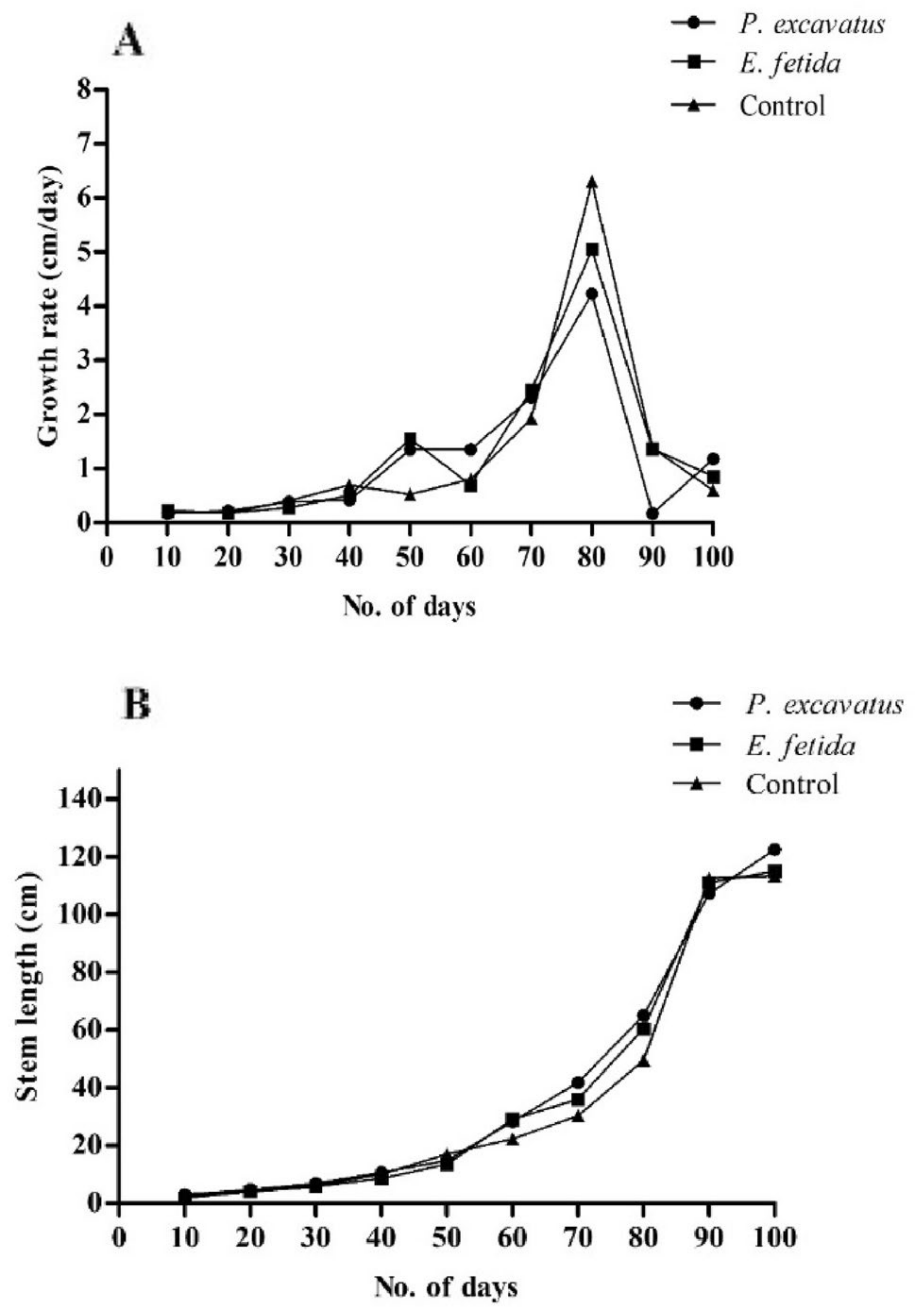
(**A**) The growth rate of *Z. mays* in the presence and absence of earthworms (**B**) Stem length of *Z. mays* under control and earthworm treatment.

**Figure 4:**
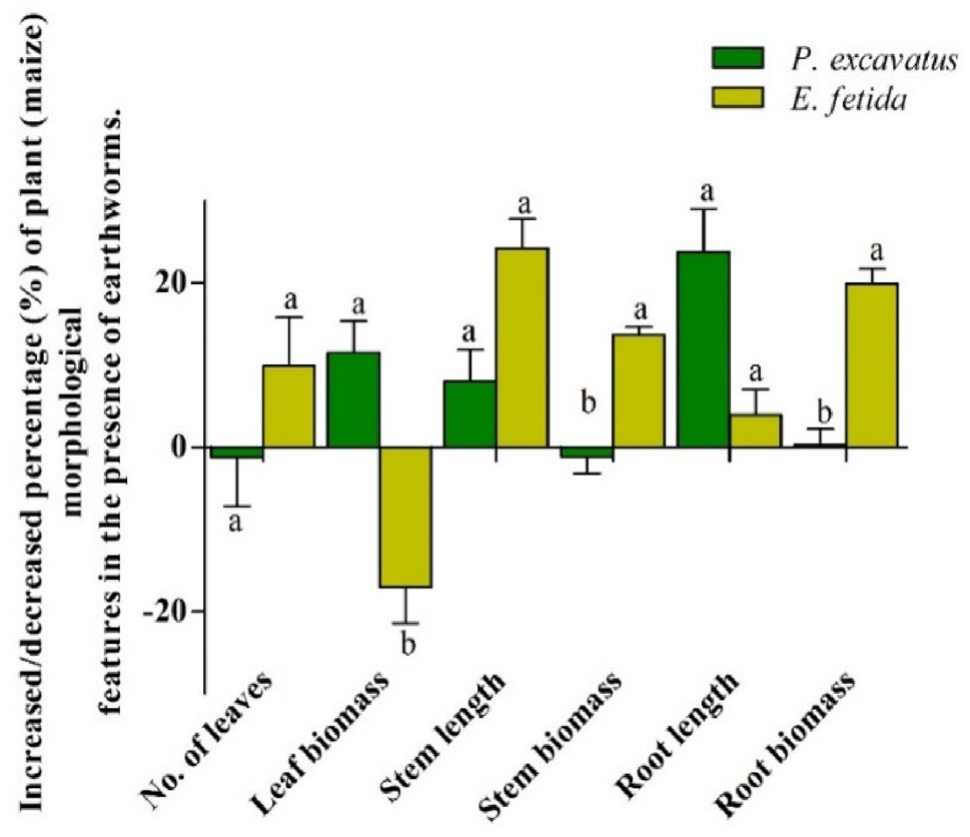
Increased/ decrease of *Z. mays* morphological characters in the presence of *P. excavatus* and *E. fetida***.** Data represent mean ± SD. Different superscripts between earthworm treatments differ significantly (*p* < 0.05) by the Tukey test at a 95% confidence level.

**Figure 5:**
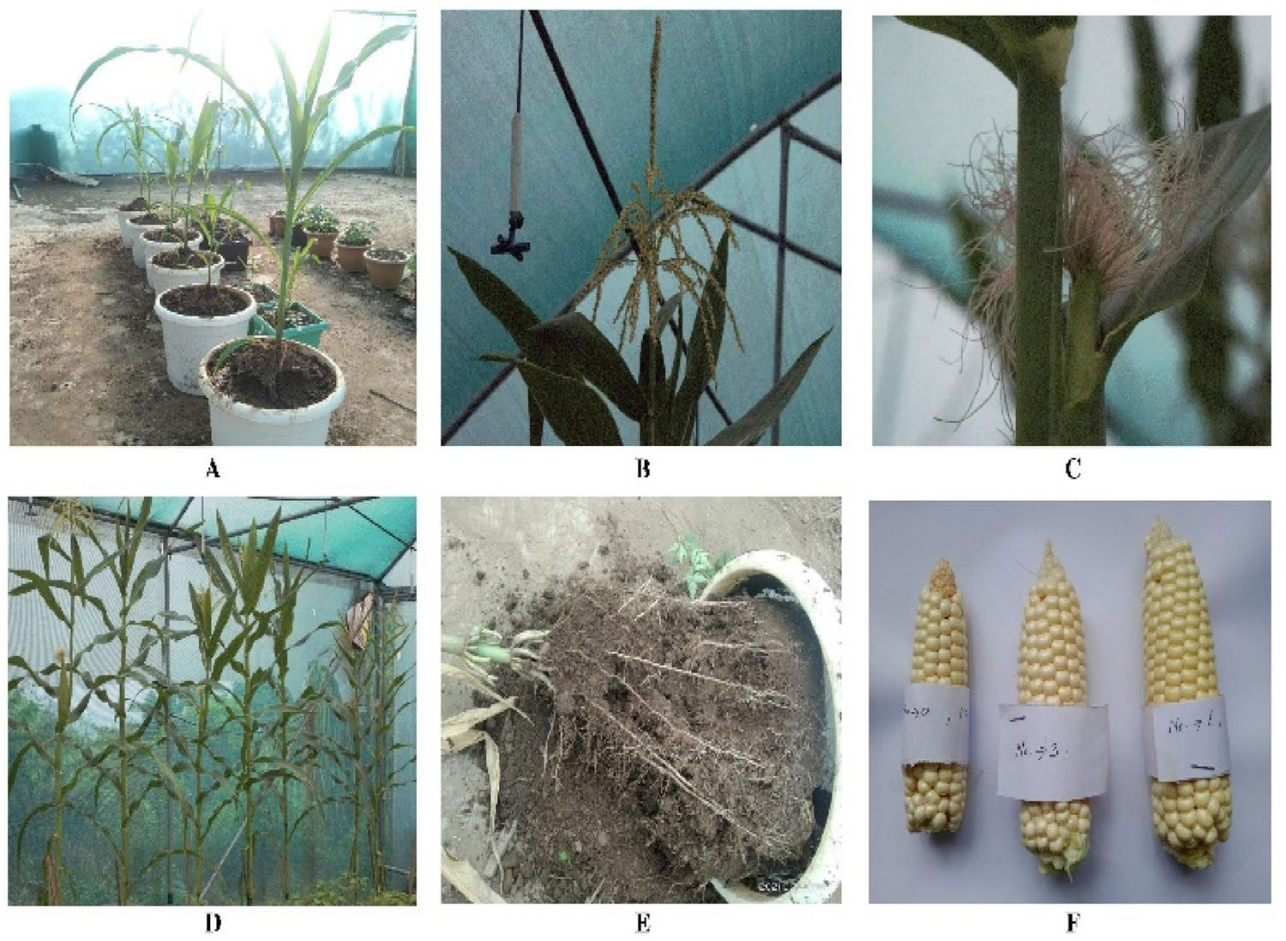
(**A**) Initial days (Day 63) of *Z. mays* grown in *P. excavatus* treated soil (**B**) Tassel bearing spikelet pairs, first observed on day 108 in *E. fetida* treated soil (**C**) First silk appears on the 110th day in *E. fetida* treated soil (**D**) Fully matured corn bearing plant as observed under greenhouse (**E**) Removing of roots from the experimented pot for plant biomass estimation and measurement of stem and root length (F) final harvested corn with healthy kernel obtained from control, *P. excavatus* and *E. fetida* treated soil.

Stem growth was maximum between 70 and 80 days, the highest average growth rate (mm/day) was recorded in *E. fetida* soil (13.6 ± 8.5), followed by *P. excavatus* (13.0 ± 5.3) and control (12.7 ± 2.5) indicating the positive effect of earthworms (Fig. 3-A). The average number of leaves per plant was maximum (14.5 ± 0.95) in *E. fetida* soil, followed by control (13.5 ± 1.29) and *P. excavatus* (13.5 ± 0.95), showing the increasing percentage of 9.93% and 6.92% in *E. fetida* and *P. excavatus* over control. Contrary to its number, the maximum leaf biomass (g) was recorded in *P. excavatus* (82.6 ± 3.5) followed by control (76.32 ± 4.45) and *E. fetida* soil (63.25 ± 4.07) (Table 2). In *P. excavatus* treated soil, leaf biomass was 11.52% higher than in control, while in *E. fetida*, biomass was 16.42% lower (Fig. 4)*.* With the broader leaf diameter, plants were found to be healthy, and no infestation of plants by insects was observed as the experiment was conducted in a greenhouse (Fig. 5A-F). Also, unlike *C. chinense*, no pathogenic infections occurred in the plants; all the seedlings grown, matured, and successfully bore corn of varying sizes, depending on the treatments. The average stem length (Fig. 3B) at the final harvest was maximum in *E. fetida* (246 ± 19.05), followed by *P. excavatus* (222 ± 12.28) and control (206 ± 13.22), exhibiting an increasing trend of 19.24% and 7.82%, respectively, over the control. Maximum stem biomass was recorded in *E. fetida*(592.16 ± 7.06), followed by *P. excavatus* (517.24 ± 15.44) and control (517.71 ± 7.12) showing significant differences (*F*_(2, 11)_ = 79.78, *p* < 0.05). Multiple comparisons test shows that the mean stem biomass from *E. fetida* was significantly (p < 0.05) higher than from *P. excavatus* and control pot. Depending on the treatment, root length also differs significantly (*F*_(2,13)_ = 21.41, *p* < 0.05), where the maximum was observed in *P. excavatus*(87.9 ± 5.43), followed by *E. fetida* (69.45 ± 4.1) and control (68.15 ± 1.95) (Table 2). Multiple comparisons test indicates that the mean root length from *P. excavatus* was significantly higher than that of *E. fetida* and control. In *P. excavatus,* a 28.9% increase in root length over the control was observed, while in *E. fetida,* root length was increased by only 1.9% (Fig. 4).

**Table 2:** Morphological characteristics of *Z. mays* plants in the presence and absence of earthworm.

Treatment	No. of leaves	Leaf biomass (g)	Stem length (cm)	Stem biomass (g)	Root length(cm)	Root biomass (g)
*P. excavatus*	13.5 ± 0.95^a^	82.6 ± 3.51^a^	222 ± 12.28^a^	517.24 ± 15.44^a^	87.9 ± 5.43^a^	107.55 ± 4.84^b^
*E. fetida*	14.5 ± 0.95^a^	63.25 ± 4.07^b^	246 ± 19.05^a^	592.16 ± 7.06^b^	69.45 ± 4.1^b^	126.49 ± 5.69^a^
Control	13.5 ± 1.29^a^	76.32 ± 4.45^ab^	206 ± 13.22^a^	517.71 ± 7.12^a^	68.15 ± 1.95^b^	104.59 ± 4.81^b^
*F*	2.21	23.07	14.13	79.78	21.41	30.11
*p*-value	0.16	0.0002*	0001*	2.8321E-7*	0.000077*	0.000035*

Data represent mean ± SD. * indicate a significant difference at *p* < 0.05. Mean with different superscripts within the same row differ significantly (*p* < 0.05) by the Tukey test at a 95% confidence level.

A significant variation of root biomass (*F*_(2, 11)_ = 30.11, *p* < 0.05) was also recorded with a maximum in *E. fetida* treated soil (126.49 ± 5.69) followed by *P. excavatus* (107.55 ± 4.84) and control (104.59 ± 4.81), having shown the increasing trend of 20.93% and 2.82% respectively over control. Post hoc test shows root biomass from earthworm-treated soil was significantly (*p* < 0.05) higher compared to control, however, no significant differences (*p* > 0.05) were observed between the two earthworm species. Among the treatments, the total kernel count per corn was significantly different (*F*_(2, 9)_ = 37.78, *p* < 0.05). The highest number of kernels was present in *P. excavatus* (333.5 ± 13.5), followed by *E. fetida* (261.5 ± 16.5) and control (235 ± 22), showing 41.91% and 11.27% increase over control. Similarly, there was also a significant difference (*F*_(2, 9)_ = 7.92, *p* < 0.05) on average kernel weight with maximum production from *P. excavatus* (104.6 ± 14.9) followed by *E. fetida* (66.68 ± 6.78) worked soil and control (56.32 ± 10.38) exhibiting 95.05% and 24.34% increase over control.

### Earthworm effects on soil nutrients

#### Soil pH

**Figure 6:**
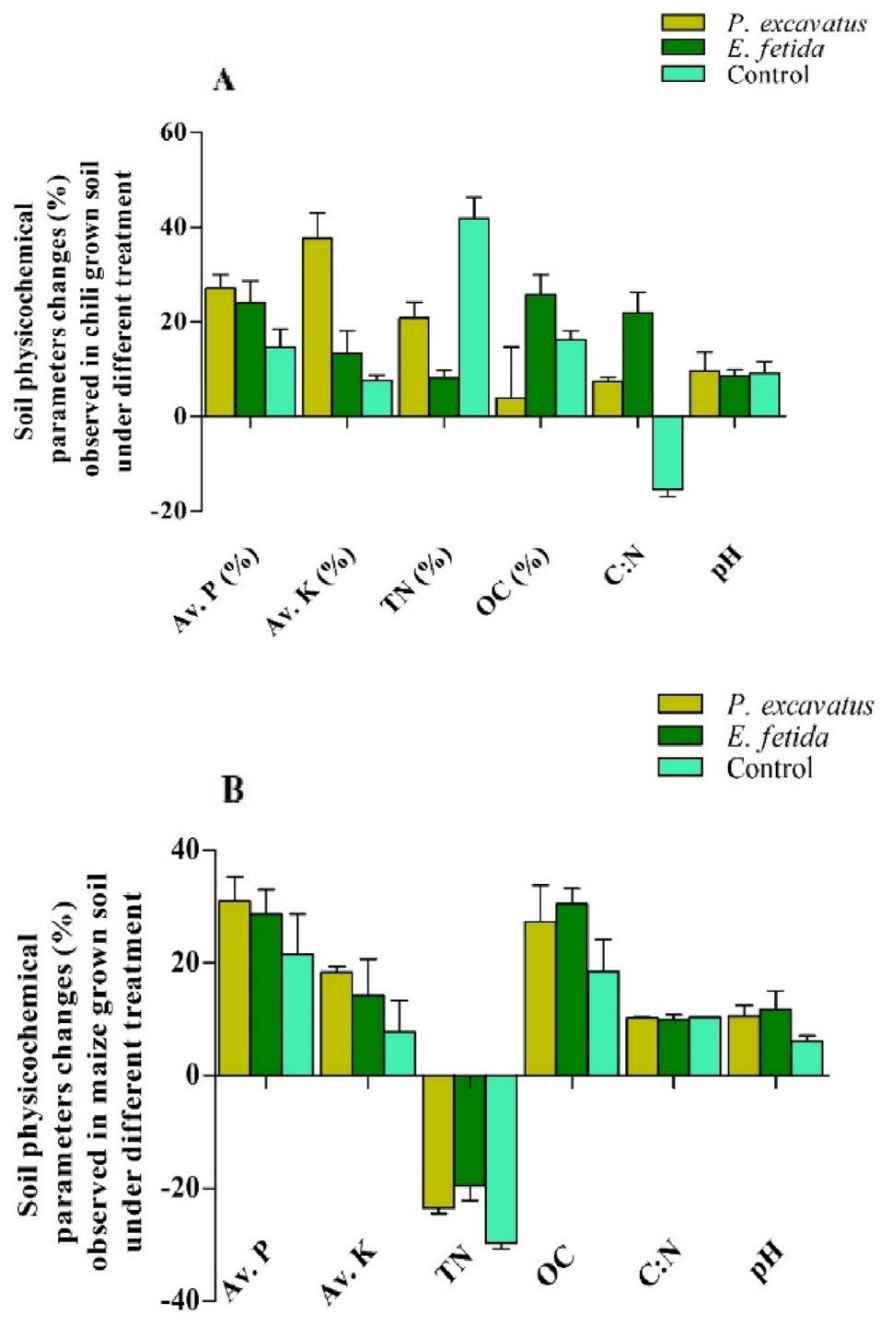
Soil physicochemical percentage changes over initial value in *C. chinense* (**A**), *Z. mays* (**B**) grown pot in the presence and absence of earthworm.

Changes observed in various physicochemical parameters such as pH, OC, TN, P, and K reflect the effects of earthworms on soil nutrient mineralization. Initially, soil pH was slightly acidic (5.6 ± 0.04), but at the end of the experiment, it increased marginally in both *C. chinense* and *Z. mays*-grown soil in the presence of *E. fetida, P. excavatus*, and control (Table 3). In *C. chinense* grown soil, in the presence of *P. excavatus, E. fetida*, and control, the final pH was 6.14 ± 0.34, 6.09 ± 0.15, and 6.12 ± 0.28 with an increased percentage of 9.6%, 8.5%, and 9.18% respectively (Fig. 6A). In *Z. mays* soil, the final soil pH was 5.79 ± 0.65, 6.26 ± 0.26, and 5.95 ± 0.08 in the *P. excavatus and E. fetida* soil and control with an increasing percentage of 3.30%, 11.73%, and 6.12% respectively (Fig. 6B). The increase in soil pH was the least affected by the earthworm's activities and did not vary significantly (*F*_(2, 6)_ = 0.03, *p* > 0.05) among different treatments.

**Table 3:** Initial and final physicochemical characteristics of soil obtained from *Z. mays*and *C. chinense*grew pot treated with *P. excavatus*, *E. fetida*, and control.

*C. chinense*						
	pH	OC (%)	TN (%)	C:N	P (mg/kg)	K (mg/kg)
Initial	5.6 ± 0.04^a^	2.63 ± 0.25^b^	0.42 ± 0.02^a^	6.17 ± 0.62^a^	33.15 ± 2.31^c^	185.75 ± 18.11^b^
*P. excavatus*	6.14 ± 0.34^a^	3.24 ± 0.24^ab^	0.51 ± 0.04^ab^	6.56 ± 1.02^a^	41.96 ± 3.84^a^	263.16 ± 15.39^a^
*E. fetida*	6.09 ± 0.15^a^	3.29 ± 0.32^a^	0.46 ± 0.03^ab^	7.42 ± 1.17^a^	41.03 ± 1.00^ab^	208.53 ± 9.75^ab^
control	6.12 ± 0.28^a^	3.04 ± 0.06^b^	0.6 ± 0.04^b^	5.17 ± 0.62^a^	37.93 ± 1.15^ab^	202.88 ± 12.76^ab^
*F*	3.43	4.61	13.71	3.27	8.41	21.78
*p*-value	0.07	0.03*	0.002*	0.08	0.007*	0.000038*

*Z. mays*						
	pH	OC (%)	TN (%)	C:N	P (mg/kg)	K (mg/kg)
Initial	5.6 ± 0.04^a^	2.63 ± 0.25^b^	0.42 ± 0.02^b^	6.17 ± 0.62^b^	33.15 ± 2.31^c^	185.75 ± 18.11^b^
*P. excavatus*	5.79 ± 0.65^a^	3.34 ± 0.22^ab^	0.32 ± 0.01^a^	10.21 ± 0.21^a^	43.38 ± 2.85^a^	218.63 ± 7.34^a^
*E. fetida*	6.26 ± 0.26^a^	3.4 ± 0.38^a^	0.34 ± 0.02^a^	9.94 ± 1.41^a^	42.54 ± 0.61^ab^	215.68 ± 4.53^ab^
control	5.95 ± 0.08^a^	3.11 ± 0.2^ab^	0.3 ± 0.02^a^	10.35 ± 0.04^a^	40.19 ± 1.53^bc^	206.46 ± 9.95^ab^
*F*	1.84	4.81	23.9	19.6	16.4	7.03
*p*-value	0.21	0.03*	0.000239*	0.000481*	0.001*	0.006*

Data represent mean ± SD. * indicate a significant difference at *p* < 0.05. Mean with different superscripts within the same row differ significantly (*p* < 0.05) by the Tukey test at a 95% confidence level.

#### Organic carbon (%)

Initially, the OC of the soil samples used for the experiment was 2.63 ± 0.25 (Table 3). At the end of the experiment, OC increased to 3.04 ± 0.06 to 3.4 ± 0.38 depending on the plant grown and the presence and absence of earthworms. In *C. chinense* grown soil, the maximum amount of OC was present in the presence of *E. fetida* (3.29 ± 0.32), followed by *P. excavatus* (3.24 ± 0.24) and control (3.04 ± 0.06) with 25.76%, 23.47%, and 16.24% increase over the initial concentration (Fig. 6A). Similarly, in *Z. mays*-grown soil, the highest concentration of OC was recorded in the presence of *E. fetida* (3.4 ± 0.38)*, P. excavatus* (3.34 ± 0.22)*,* and control (3.1 ± 0.2) with 30.48% 27.36% and 18.4% increase (Fig. 6B).

#### TN (%)

Contrary to other parameters, total nitrogen showed an uneven increase and decrease. Initially, the average amount of TN was 0.42 ± 0.02; however, at the end of the experiment, in *C. chinense*-grown soil, TN was increased to 0.51 ± 0.04 and 0.46 ± 0.03 in the presence of *P. excavatus* and *E. fetida,* respectively. While in control, the final concentration of TN was increased to 0.6 ± 0.04. Variations of TN among the treatment were significantly (*F*_(2, 6)_ = 9.9, *p* < 0.05) different. Multiple comparisons test showed TN in *P. excavatus* and *E. fetida* were insignificant (*p* > 0.05), but the control was significantly (*p* < 0.05) lower compared to *E. fetida*. Depending on the presence and absence of earthworms, the increased percentage of TN over the initial value were significantly (*F*_(2,6)_ = 25.61, *p* = 0.001) different with the highest being observed in control (41.86%), *P. excavatus* (20.81%), and *E. fetida* (8.19%) (Fig. 6A).Post hoc test shows TN increased in the control pot was significantly (*p* < 0.05) higher compared to *P. excavatus* and *E. fetida.* While in *Z. mays*-grown soil, TN decreased (Table 3) in all the treatments, and the final concentration was 0.32 ± 0.01, 0.34 ± 0.02, and 0.3 ± 0.02 in *P. excavatus*, *E. fetida*, and control, respectively. The maximum reduction was recorded in control (29.72%), followed by *P. excavatus* (23.42%), and *E. fetida* (19.48%) (Fig. 6B).

#### C:N

C:N ratio is another essential parameter to indicate the nutrient stability of the soil. C:N ratio 15–20 is considered an acceptable range for agronomy (FAO, 2020). In the present study, substantial variations of C:N were observed depending on the treatment but well within the suggested required range. Having the initial ratio of 6.17 ± 0.62 in the *C. chinense* grown soil, C:N ratio in the presence of *E. fetida* and *P. excavatus* were increased to 7.42 ± 1.17 and 6.56 ± 1.02 with 21.92% and 7.48% increase. While in control, the final C:N ratio was decreased by 15.39% (5.17 ± 0.62). However, in *Z. mays* grown-soil, C:N ratio was 10.35 ± 0.34, 10.21 ± 0.21, and 9.94 ± 0.41 in control, *P. excavatus*, and *E. fetida*, respectively exhibiting a maximum increase of C:N ratio in control (68.68%), followed by *P. excavatus* (66.58%), and *E. fetida* (63.46%). Analysis of variance study shows that in *Z. mays* grown soil, the increased percentage of C:N varies significantly (*F*_(2, 6)_ = 47.17, *p* < 0.05) among treatments.

#### Phosphorus (P) (mg/kg)

In *C. chinense-*grown soil, although no significant (*F*_(2, 6)_ = 2.33, *p* > 0.05) differences were observed among the treatment, P was increased to 41.96 ± 3.84, 41.03 ± 1.00, and 37.93 ± 1.15 in the presence of *P. excavatus, E. fetida*, and control, respectively. The increased percentage of P in *P. excavatus, E. fetida*, and control were 31.03%, 28.67%, and 21.59% (Fig. 6A). Similarly, in *Z. mays-*grown soil, a higher percentage of increase was recorded in the presence of *P. excavatus, E. fetida,* and control, respectively, and the final concentration of P was 43.38 ± 2.85, 42.54 ± 0.61, 40.19 ± 3.10.

#### Potassium (K) (mg/kg)

The initial amount of K was 33.15 ± 2.31 (Table 3). In *C. chinense*-grown soil, K was increased at the final and showed significant differences (*F*_(2, 9)_ = 26.86, *p* < 0.05) among the treatment. The highest concentration was present in *P. excavatus* (263.16 ± 15.39), *E. fetida* (208.53 ± 9.75), and control (202.88 ± 12.76), with increasing trend of 41.67%, 12.26%, and 9.22%, respectively over the initial value. Increased percentage of K in treatment also shows significant differences (*F*_(2,6)_ = 15.23, *p* < 0.05). The post hoc test shows an K increased in *P. excavatus* inoculated soil was significantly (*p* < 0.05) higher compared to *E. fetida* and control. In *Z. mays*-grown soil also, although the final amount of K varies insignificantly among the treatment (*F*_(2,9)_ = 2.78, *p* > 0.05), substantially higher amount of K concentration was recorded in the presence of earthworms *P. excavatus* (218.63 ± 7.34), *E. fetida* (215.68 ± 4.53), and control- 206.46 ± 9.95). In *P. excavatus* inoculated soil, K was increased by 18.44% over the initial value, while in *E. fetida* and control, 14.28%, and 7.75% increase over the initial concentration was observed.

## Discussion

Sustainable agriculture encompasses food production from plants or animals using different techniques without adverse impacts on humans, the environment, and animals. Extensive use of fertilizers and pesticides boosts food production but also deteriorates the biodiversity (above and below the ground) associated with cropland. As an essential component of the soil, earthworms maintain soil fertility and play a key role in sustainability. The presence of earthworms enhanced the soil nutrient, plant biomass (leaves, stem, root), and fruit yield (ripened chili, kernel count, and kernel weight), which was consistent with the notion that earthworms increased plant growth^13^. In the present findings, plants grown in earthworm treatment *(P. excavatus* and *E. fetida*) resulted in better morphological characteristics such as shoot biomass, stem length, number of leaves, coloration, biomass, and fruit yield.In *P. excavatus* treated soil, *C. chinense* growth rate was 11.43% higher. However, in *E. fetida*inoculated soil, *C. chinense*resulted in a lower growth rate (-9.65%), stem length (-2.77%), stem biomass (-16.43%) and root length (-2.42%). The average growth rate of *Z. mays* was higher in *E. fetida* (13.6 ± 8.5 mm/day), and *P. excavatus* (13.0 ± 5.3 mm/day) than in control (12.7 ± 2.5 mm/day).

**Figure 7:**
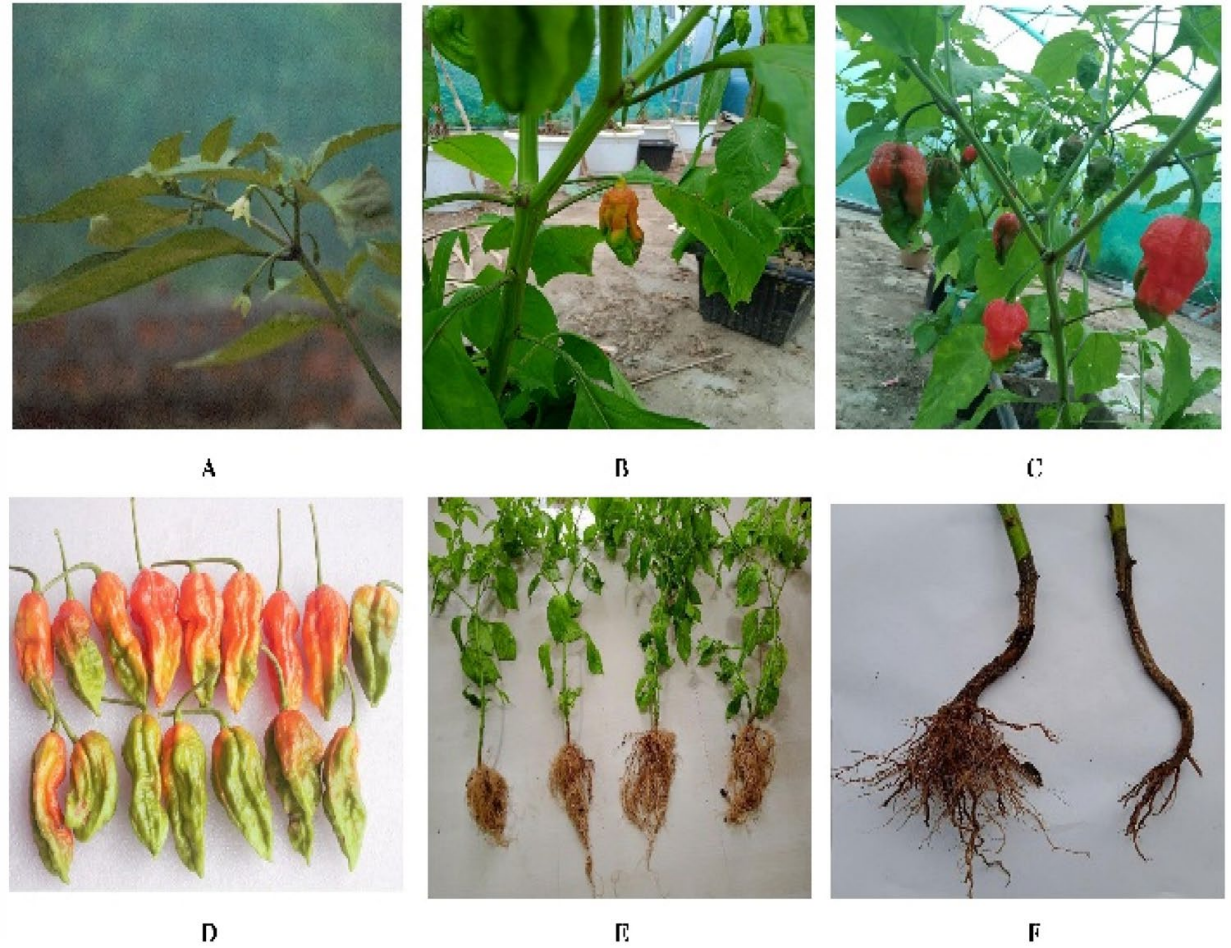
(**A**)Flowering of *C. chinense*observed on day 50 in *P. excavatus* treated soil (**B**) Ripen fruit from *P. excavatus* treated soil observed on 78th day (**C**) Fully ripen chili in*E. fetida* treated soil (**D**) One-time harvest from single plant grown in *P. excavatus* treated soil (**E**) Freshly harvested plant taken for root length and biomass estimation (**F**) Fungal infected root and stem causing rotting of plants starting from underground roots to stem resulting into the black in color, observed in *E. fetida* treated soil.

It was observed that during the initial 30–35 days, plants grew successfully in the earthworm treated soil and control. However, due to fungal infection, 50% of the plant roots in *E. fetida* inoculated soil (Fig. 7A–F) did not survive till maturity, and the average growth rate was negatively affected (Fig. 7F). In *P. excavatus* soil, 100% of plants survived, and no fungal infections were observed. Therefore, it is less likely that earthworm-associated pathogens cause disease in plants. Fungal infections could be due to excess waterlogging in the root, depriving the roots of oxygen making them more susceptible to infections. Also, unsterilized seeds may be the cause of fungal infection in the *E. fetida* inoculated soil.Other researchers have also reported that, *C. chinense* is susceptible to various fungal diseases, including *Colletotrichum capsici*, *Colletotrichum gloeosporoides*, *Sclerotinia sclerotiorum*, *Rhizoctonia solani*, and *Corynespora cassicola*^24^. In *C. chinense*, due to fungal infection total fruit harvest, plant growth rate, leaf biomass, and root biomass were lowered. Apart from the microbial effects, concentrations of salts in the soil, mainly NaCl, are also responsible for the reduction in the productivity of economically important crops such as chili (*C. annuum*), tomato (*Solanumlycopersicum*), and potato (*S. tuberosum*)^18,25^. Also,Aktaş et al. (2006)^26^and Niu and Cabrera (2010)^27^considered chili plants to be very susceptible to this abiotic factor.

Researcher in the past (Xiao, Z. et al*.* 2018)^28^ also reported that the presence of earthworms increased plant growth by 20% and further emphasized that earthworms' effect on plant growth is more effective when mixed earthworm species are inoculated at the same time. In the present study, distinct differences in leaf coloration were also observed, with leaves acquiring dark green color in earthworm-worked soil, which could be due to more chlorophyll and carotenoid content^29^. Brown et al. (2004)^15^suggested that there are five possible ways through which earthworms positively affect plant productivity. (i) Biocontrol of pests and diseases; (ii) stimulation of plant microbial associations (iii) production of plant growth regulating substances (iv) changes in soil physicochemical structures, (v) and enhanced nutrient availability for absorption through roots. In *C. chinense*, the effects of *P. excavatus* were higher, as evident from the increasing number of leaves (250.4%), leaf biomass (92%), stem length (11.6%), stem biomass (41.77%), root length (35%) and root biomass (43.15%) over the control.Van Groenigen et al. (2014)^30^ reported that the presence of earthworms increased the aboveground plant biomass productivity by 23% and suggested that earthworms enhanced plants' growth mainly through their ability to release nitrogen trapped in organic matter. Similarly, Trap et al. (2021)^31^ also reported that the presence of endogeic earthworms significantly increased the shoot biomass (26%) of rice (*Oryza sativa*). Increased plant biomass in the presence of earthworms is attributed to releasing inaccessible forms of nutrients in the soil, making it available for plant absorption. Along with the other plants' morphological characteristics, the present study has shown the positive effect of earthworm (*P. excavatus*), recording 71.3% higher chili fruit production per plant. Gong and Gao (2019)^32^ opined that shoot biomass is less sensitive to changes in soil fertility.Van Groenigen (2013)^33^reported that once earthworms processed soils, their effects on plant growth get reduced. Similarly, earthworms' role in plant growth enhancement is more impactful in developing soil than in developed soil and further emphasized that during early to a late succession of plants community, earthworm activities play a significant role^12^. Barrion and Litsinger (1997)^34^ reported that at very high density (17,294 ind./m^2^) *Dichogaster* *curgensis* Michaelsen becomes a pest in the irrigated rice field, causing leaks in rice levees, injured roots, uneven growth, and seedling death. However, in the present study, *P. excavatus* and *E. fetida,* surface dweller were used for the experiment, and naturally, these species has not been recorded at such high density. Further, due to low density of earthworm the species did not revealed any negative effects on plant growth.

In the present study, both the earthworm species have a fair share of effects on *Z. mays*, with *P. excavatus* more perceptible in leaf biomass (11.52%) and root length (28.9%). At the same time, *E. fetida* effects were more in morphological characters, such as the number of leaves (9.93%), stem length (24.63%), stem biomass (14.39%), and root biomass (20.93%). The effect on kernel count and weight was more with *P. excavatus* (41.91% and 95.07%) than *E. fetida* (11.27% and 24.35%). The bacteria such as *Bacillus safeness, Bacillus flexus,* and *Staphylococcus haemolyticus*associated with earthworm gut show plant growth-promoting potentials with the production of indole acidic acid (IAA), gibberellic acid (GA), ammonia, aminocyclopropane-1-carboxylic acid (ACC) deaminase activity, and phosphate solubilizing activities^35^. The higher percentage of plant growth characteristics in the presence of earthworms could be attributed to associated bacteria that release the insoluble form of soil nutrients into a soluble form, making it available for plants to absorb^36,37^. Irrespective of species, the presence of earthworm increases the microbiota that improved soil nutrient content (N, P, K) available for plant absorption. Plant growth hormones such IAA secreted by earthworm associated bacteria stimulates the plant cell elongation or divisions, especially in roots, thereby providing greater surface area for soil nutrient absorption and enhancing root growth and length. However, the mechanism connecting earthworms-soil promoting plants' growth response is more complex due to the involvement of multiple factors interaction^38^.

P in the soil was increased by 27.12% (*P. excavatus*) and 24.11% (*E. fetida*), while in *Z. mays*-grown soil, 31.03% (*P. excavatus*) and 28.67% (*E. fetida*) increase were observed. In *C. chinense*-grown soil, K was observed to increase by 42.39% (*P. excavatus*) and 12.70% (*E. fetida*). While in *Z. mays*-grown soil, an 18.38% and 14.27% increase was observed. The increased concentration of phosphorus in the earthworm-worked soil may be attributed to earthworm activity conducive to phosphate-dissolving bacteria in the soil^39^. The activity of earthworm gut enzymes, phosphatase, formation of organic acids, and discharge of total phosphorus from a complex form of humic acid mediated by microbial activity might contribute to the increased concentration of P in the soil^40,41^. Many earthworm gut-associated bacteria are reported to have phosphate and potassium-solubilizing bacteria. Yakkou et al. (2022)^42^ observed that out of 16 bacteria isolated from earthworm gut, six bacteria, namely *Pseudomonas aeruginosa*, *Pantoea vagans, Buttiauxella gaviniae, Raoultella planticola, Aeromonas* sp*. Aeromonas drosophila* has the potential to solubilize insoluble forms of Potassium. Therefore, apart from the other biochemical process, an increased P and K could be attributed to earthworm-associated bacteria that solubilize the bound form of macronutrients^43^.

Irrespective of earthworm species, the increased amount of macronutrients in the soil is more distinct, indicating its role in nutrient turnover, making it more readily available for plant absorption. An increase in organic carbon and inconsistent changes in total nitrogen (Table 3) might be attributed to the decomposition of leaves from plants themselves. Plant-available water, water-holding capacity, bulk density, and soil organic matter were also reported to increase earthworm presence^10^. Zhao et al. (2013)^44^ reported an 11% increase in nitrogen in the presence of earthworms. Enhanced plant productivity increases the organic matter input to the soil, increasing the earthworm's food supply.Earthworms’ effects on the soil and plants are influenced by various factors, including their feeding preferences, soil types, and plant species-specific traits. Variations in plant root systems also impact earthworm presence, seeking areas with suitable structures. Moreover, earthworms' interactions with soil microorganisms, influenced by different plant species, also impact their behavior and health of the above-ground productivity.

Despite higher soil fertility in earthworm-treated soil, a lack of significant differences in *Z. mays* stems length and its biomass from earthworm-treated soil and control were observed (Table 2). Also, for *C. chinense*, insignificant differences were observed in stem length from earthworm-treated soil and control. It is important to note that our study was short-term, conditioned for optimum earthworm activities through proper moisture maintenance, and conducted under greenhouse conditions. Earthworm affects plant biomass productivity more in soil with no earthworm legacy than in earthworm-mediated soil. Therefore, it is essential to elucidate the long-term effects of earthworms on soil physicochemical parameters, plant productivity, and associated microorganisms that bring about various changes in soil systems in many different natural environments.

## Conclusion

As macroinvertebrates, *P. excavatus* and *E. fetida* demonstrated promising abilities in enhancing plant biomass and soil nutrient content. Explicitly, the influence of *P. excavatus* was more pronounced in *C. chinense*, as evidenced by the increased number of leaves, biomass, stem length, root length, root biomass, and fruit harvest. Conversely, *E. fetida* significantly impacted *Z. mays*, leading to enhanced stem length, stem biomass, root biomass, number of leaves, kernel count, and kernel weight. The presence of earthworm also increased soil nutrients, further supporting improved plant morphological characteristics. Consequently, the study did not identify a superior earthworm species based on their effects on plant productivity. Nonetheless, these findings suggest that organic cultivation of seasonal vegetables, without synthetic fertilizers, can be achieved by introducing earthworms. This approach becomes particularly valuable for kitchen gardens in urban areas with limited space for plant growth. Using earthworms as mediators for fulfilling soil nutrient requirements, even miniature plants like *C. chinense* can be cultivated purely organically.

## Materials and method

### Pre-experimental preparations

Two different earthworm species were used for the experiment. (a) *E. fetida*, a commercially available species, was procured from a local vermicomposting farm, Wokha, Nagaland, India, and (b) *P. excavatus,* was sampled during an earthworm resource exploration study in Minkong forest (26°21′43.34"N and 94°33′23.42"E) under Mokokchung district, Nagaland. Both the earthworm species were mass cultured separately in the vermicomposting chamber in the Zoology Department, Nagaland University, for adaptation to the local environment and multiplication of their population. Moisture in the vermiculture setup was maintained at 60%-70% by sprinkling water regularly, and temperature ranged between 28 °C to 34.6 °C. Earthworms were fed with a mixture of urine-free pre-decomposed cow dung collected from a local farm and domestic organic waste collected from the household.

### Experimental design

Two commonly available and preferred plant species were chosen for this study *i.e. C. chinense*, locally referred to as Naga king chili, and *Z. mays*, (Maize). The plant growth experiment used loose topsoil, collected from the University campus within a depth of 0–10 cm. Prior to commencing the experiment, the seeds of *C. chinense* and *Z. mays* were sprouted by soaking them in water for 36 h.

For *C. chinense*, the experiment was conducted in triplicates using nine plastic pots with a capacity of 5 L due to the smaller size of the plants. Each pot had dimensions of 250 mm in length and 200 mm in diameter. The pots were filled with 2 mm sieved soil and water was sprinkled to maintain moisture. Subsequently, 150 g of urine-free, partially decomposed (15 days) cow dung was added to each pot as a food source for earthworms. The same amount of cow dung was applied to the control pots to ensure a uniform amount of nutrients. Since the earthworm species used were surface dwellers (epigeic), the cow dung was applied to the top layer (0–10 cm) and mixed with the soil. In the first three pots, ten healthy, clitellate individuals of *P. excavatus,* with an average weight of 350.34 ± 7.4 mg, were introduced. Similarly, ten healthy *E. fetida* individuals, with an average weight of 410.12 ± 8.5 mg, were introduced in the following three pots. Due to the smaller plant and pot size, only ten earthworm individuals were added to the pots where *C. chinense* was grown. The third set of three pots served as the control group and did not have any earthworms.

For *Z. mays*, nine plastic pots (in triplicate for each group) with a capacity of 15 L were used due to the larger size of the plants. These pots had dimensions of 390 mm in length and 320 mm in diameter. The pots were filled with 2 mm sieved soil and were regularly sprinkled to maintain optimal moisture levels. In each pot, including the control, 300 g of urine-free, partially decomposed cow dung was added as food for earthworms. Because of the larger plant and pot size, 20 healthy clitellated earthworms, *P. excavatus* (average weight 350.34 ± 7.4), and *E. fetida* (average weight 410.12 ± 8.5), were inoculated into the first and second set of pots, respectively. The remaining three pots were used as control with no earthworm.

In all the pots, the earthworms were allowed to settle and were observed for three days to ensure their normal survival and growth. Once the earthworms had settled and burrowed into the soil, a pair of sprouted *C. chinense* and *Z. mays* seeds were planted in their respective pots. The moisture level in all the experimental pots was maintained at 40–50% by regular water sprinkling. Since the experiment took place in a greenhouse, no pest attack was observed. The temperature ranged between 28 and 34.6 °C, and humidity fluctuated between 40–70%, depending on the weather conditions. Plant growth was monitored at regular intervals of 7 days (weekly) from day one until the plants complete fruiting and reached maturity. This involved measuring stem length and counting the number of leaves and fruits. In the case of *C. chinense*, once the plants began to bear fruits and mature, harvesting was conducted weekly, and the fruits were weighed to record the weekly fruit yield per plant. While for *Z. mays* kernel count and weight were done at the final harvest of the plant. The biomass of both the plants were weighted using a portable digital weighing machine (Oblivion-OBSF400A).

### Soil physicochemical analysis

Soil samples were analyzed at two intervals, i.e., before the inoculation earthworms to the pot and at the end of the pot experiment. Before analysis, mixture of soil and cow dung were collected from the pot, air-dried, sieved through a 1 mm mesh size, and kept in an airtight plastic bag. pH was measured using a digital pH meter (Lab junction-111) at a 1:20 soil–water ratio**.** Organic carbon was analyzed by a modified form of the wet oxidation initially described by Walkley and Black (1934)^45^. Total nitrogen (TN) was determined by Kel plus instrument (Pelican equipment- Classic- DX VAT-E). Phosphorus (P) was determined spectrophotometrically (Systronic spectrophotometer-166) using a modified form of Bray and Kurtz (1945)^46^. The amount of available phosphorus was determined by the intensity of blue color development when treated with a molybdate-ascorbic acid reagent. Potassium (K) was determined by flame photometer following a modified form of the ammonium acetate method described by Hanway and Heidel (1952)^47^.

### Statistical analysis

The significant differences in the number and biomass of leaves, stem, root biomass; and growth rate and total fruit harvest of both plants among the treatments were analysed using one-way ANOVA at a 95% confidence level (*p* < 0.05). Each analysis was followed up with multiple comparison tests (Tukey test) to find the mean differences between treatments. The statistical analysis was computed using SPSS (Version 22).

## Ethical declaration

1. The authors affirm that no ethical violations occurred during the collection of plants. The collection of plants was conducted in compliance with the Constitution of India (Article 371 (A)), which grants the tribal communities of Northeast India, specifically Nagaland, rightful authority to collect and utilize natural resources from non-protected regions of the state. Moreover, it is important to note that the plant species (*Capsicum chinensis* and *Zea mays*) utilized in this study are not listed under any categories of the IUCN Red List. Further, after the experiment, plant specimens were deposited in the University Herbarium with brochure numbers NU_LJ-570 and NU_LJ-571.
2. It is hereby declared that seeds of *Capsicum chinensis* (King chili) and *Zea mays* (Makai) from Zaphumi village were collected for experimental purposes in the Department of Zoology at Nagaland University, Lumami, Zunheboto. Furthermore, it is confirmed that no objections have been raised by any individual or community regarding their consent for this collection.
3. Experimental research and field studies on plants (either cultivated or wild), including the collection of plant material, comply with relevant institutional, national, and international guidelines and legislation.

## Data Availability

All data generated or analysed during this study are included in this article.

## References

[1] Shah, S., Ramanan, V. V., Singh, V. V. & Singh, K. A. Potential and prospect of plant growth promoting rhizobacteria in lentil. *Sci. Lentil Prod.* 431–451 (2018).

[2] Prescott CE (2005). Decomposition and mineralization of nutrients from litter and humus. Nutrient Acquisition by Plants.

[3] Yoshitake, S., Soutome, H. & Koizumi, H. Deposition and decomposition of cattle dung and its impact on soil properties and plant growth in a cool-temperate pasture. *Ecol. Res. ***29**, (2014).

[4] Van Groenigen JW (2019). How fertile are earthworm casts? A meta-analysis. Geoderma.

[5] Singh, J. S. & Gupta, V. K. Soil microbial biomass: a key soil driver in management of ecosystem functioning. *Sci. Total Environ. ***634**, (2018). 10.1016/j.scitotenv.2018.03.373 29635193

[6] Wurst, S., Sonnemann, I. & Zaller, J. G. Soil Macro-invertebrates: their impact on plants and associated aboveground communities in temperate regions. in (2018). doi: 10.1007/978-3-319-91614-9_8.

[7] Zaller, J. G. et al. Subsurface earthworm casts can be important soil microsites specifically influencing the growth of grassland plants. *Biol Fertility of Soils ***49**, (2013). 10.1007/s00374-013-0808-4 PMC4459550 26069355

[8] Fahey, T. J. et al. Earthworm effects on the incorporation of litter C and N into soil organic matter in a sugar maple forest. *Ecol Appl ***23**, (2013). 10.1890/12-1760.1 23967585

[9] Sharma DK, Tomar S, Chakraborty D (2017). Role of earthworm in improving soil structure and functioning. Current Sci..

[10] Hallam, J. et al. Effect of earthworms on soil physico-hydraulic and chemical properties, herbage production, and wheat growth on arable land converted to ley. *Sci. Total Environ. ***713**, (2020). 10.1016/j.scitotenv.2019.136491 31962242

[11] Frelich, L. E. et al. Earthworm invasion into previously earthworm-free temperate and boreal forests. *Biol. Invasions* vol. 8 (2006).

[12] Mudrák, O. & Frouz, J. Earthworms increase plant biomass more in soil with no earthworm legacy than in earthworm-mediated soil, and favour late successional species in competition. *Func. Ecol ***32**, (2018).

[13] Scheu, S. Effects of earthworms on plant growth: Patterns and perspectives. in *Pedobiologia* vol. 47 (2003).

[14] Paliwal, P. Exploration study of organic manure and earthworm ( Pheretima posthuma ) inoculation on quality of coal mine soil. **2**, 75–77 (2020).

[15] Brown GG, Edwards CA, Brussaard L (2004). How earthworms affect plant growth: Burrowing into the mechanisms. Earthworm Ecol. Second Edition..

[16] Brown, G. G. et al. Effects of earthworms on plant production in the tropics. *Earthworm management in tropical agroecosystems* (1999).

[17] Edwards, C. A. & Arancon, N. Q. Earthworms, Soil Structure, Fertility, and Productivity BT - Biology and Ecology of Earthworms. in (eds. Edwards, C. A. & Arancon, N. Q.) 303–334 (Springer US, 2022). doi:10.1007/978-0-387-74943-3_10.

[18] Medina-Lara, F. *et al.* Red and brown soils increase the development and content of nutrients in habanero pepper subjected to irrigation water with high electrical conductivity. *HortScience ***54**, (2019).

[19] Mondal A (2020). Detoxification and eco-friendly recycling of brick kiln coal ash using Eisenia fetida: a clean approach through vermitechnology. Chemosphere.

[20] Devi J (2020). Appraisal of lignocellusoic biomass degrading potential of three earthworm species using vermireactor mediated with spent mushroom substrate: compost quality, crystallinity, and microbial community structural analysis. Sci. Total Environ..

[21] Hussain, N. et al. Metal induced non-metallothionein protein in earthworm: A new pathway for cadmium detoxification in chloragogenous tissue. *J. Hazardous Mater ***401**, (2021). 10.1016/j.jhazmat.2020.123357 32634662

[22] Das, D. et al. Eisenia fetida for vermiconversion of waste biomass of medicinal herbs: Status of nutrients and stability parameters. *Bioresource Technol ***347**, (2022). 10.1016/j.biortech.2021.126391 34838967

[23] Pottipati, S., Kundu, A. & Kalamdhad, A. S. Process optimization by combining in-vessel composting and vermicomposting of vegetable waste. *Bioresource Technol ***346**, (2022). 10.1016/j.biortech.2021.126357 34798248

[24] Talukdar, J., Saikia, A. K. & Borah, P. Survey and detection of the diseases of Bhut Jolokia ( Capsicum chinense Jacq.) in Assam. *J. Crop and Weed ***11**, 186–192 (2015).

[25] Maas, E. V. & Hoffman, G. J. Crop salt tolerance- Current assessment. *J Irrigation and Drainage Division ***103**, (1977).

[26] Aktaş, H., Abak, K., Öztürk, L. & Çakmak, I. The effect of zinc on growth and shoot concentrations of sodium and potassium in pepper plants under salinity stress. *Turkish J. Agriculture and Forestry ***30**, (2006).

[27] Niu, G. & Cabrera, R. I. Growth and physiological responses of landscape plants to saline water irrigation: a review. *HortScience ***45**, (2010).

[28] Xiao, Z. *et al.* Earthworms affect plant growth and resistance against herbivores: a meta-analysis. *Functional Ecol ***32**, (2018).

[29] Usmani, Z. et al*.* Enhanced soil fertility, plant growth promotion and microbial enzymatic activities of vermicomposted fly ash. *Sci. Rep. ***9**, (2019). 10.1038/s41598-019-46821-5 PMC6639538 31320739

[30] Van Groenigen JW (2014). Earthworms increase plant production: a meta-analysis. Sci. Rep..

[31] Trap J (2021). Effects of the earthworm Pontoscolex corethrurus on rice P nutrition and plant-available soil P in a tropical Ferralsol. Appl. Soil. Ecol..

[32] Gong, H. & Gao, J. Soil and climatic drivers of plant SLA (specific leaf area). *Global Ecol Conserv ***20**, (2019).

[33] Roubíčková A, Mudrák O, Frouz J (2013). Soil fauna plant interactions during succession at post-mining sites. Soil Biota and Ecosyst. Develop. Post Mining Sites.

[34] Barrion AT, Litsinger JA (1997). Dichogaster nr. curgensis Michaelsen (Annelida: Octochaetidae): an earthworm pest of terraced rice in the Philippine Cordilleras. Crop Protection.

[35] Banerjee, A. *et al.* Enteric bacteria from the earthworm (Metaphire posthuma) promote plant growth and remediate toxic trace elements. *J. Environ. Manag. ***250**, (2019). 10.1016/j.jenvman.2019.109530 31521922

[36] Biswas JK (2018). Potential application of selected metal resistant phosphate solubilizing bacteria isolated from the gut of earthworm (Metaphire posthuma) in plant growth promotion. Geoderma.

[37] Houida S (2022). Biopriming of maize seeds with plant growth-promoting bacteria isolated from the earthworm Aporrectodea molleri: effect on seed germination and seedling growth. Lett. Appl. Microbiol..

[38] Braga, L. P. P. *et al.* Disentangling the influence of earthworms in sugarcane rhizosphere. *Sci. Rep. ***6**, (2016). 10.1038/srep38923 PMC5156904 27976685

[39] Ramnarain YI, Ansari AA, Ori L (2019). Vermicomposting of different organic materials using the epigeic earthworm Eisenia foetida. Int. J. Recycling of Organic Waste in Agriculture.

[40] Gusain R, Suthar S (2020). Vermicomposting of duckweed (Spirodela polyrhiza) by employing Eisenia fetida: changes in nutrient contents, microbial enzyme activities and earthworm biodynamics. Biores. Technol..

[41] Sharma K, Garg VK (2018). Vermicomposting: a green technology for organic waste management. Energy, Environ, Sustain..

[42] Yakkou L (2022). Assessment of earthworm (Aporrectodea molleri)’s coelomic fluid-associated bacteria on different plant growth-promoting traits and maize germination and seedling growth. Biocatal. Agric. Biotechnol..

[43] Bhakta JN, Sarkar B, Brahma P (2022). Isolation and characterization of potential phosphate solubilizing bacteria from earthworm (Metaphire posthuma) for applying as biofertilizer. Org. Agric..

[44] Zhao C (2018). Insights into the role of earthworms on the optimization of microbial community structure during vermicomposting of sewage sludge by PLFA analysis. Waste Manag..

[45] Walkley, A. & Black, I. A. An examination of the degtjareff method for determining soil organic matter, and a proposed modification of the chromic acid titration method. *Soil Sci. ***37**, (1934).

[46] Bray, R. H. & Kurtz, L. T. Determination of total, organic, and available forms of phosphorus in soils. *Soil Sci. ***59**, (1945).

[47] Hanway, J. & Heidel. *Soil analysis methods as used in Iowa state college soil testing laboratory*. *Plant and Soil* (1952).

